# A nomogram prediction model for lymph node metastasis in endometrial cancer patients

**DOI:** 10.1186/s12885-021-08466-4

**Published:** 2021-06-29

**Authors:** Zhiling Wang, Shuo Zhang, Yifei Ma, Wenhui Li, Jiguang Tian, Ting Liu

**Affiliations:** 1 Department of Obstetrics and Gynecology, Qilu Hospital of Shandong University, 107 Wenhua Xi Road, Jinan, Shandong Province 250012 P. R. China; 2 Department of Obstetrics and Gynecology, Jinan Central Hospital Affiliated to Shandong University, Jinan, 250013 Shandong Province China; 3 Department of Emergency, Qilu Hospital of Shandong University, Jinan, Shandong Province China

**Keywords:** Endometrial cancer, Lymph node metastasis, Nomogram, Lymphadenectomy

## Abstract

**Background:**

This study aimed to explore the risk factors for lymph node metastasis (LNM) in patients with endometrial cancer (EC) and develop a clinically useful nomogram based on clinicopathological parameters to predict it.

**Methods:**

Clinical information of patients who underwent staging surgery for EC was abstracted from Qilu Hospital of Shandong University from January 1st, 2005 to June 31st, 2019. Parameters including patient-related, tumor-related, and preoperative hematologic examination-related were analyzed by univariate and multivariate logistic regression to determine the correlation with LNM. A nomogram based on the multivariate results was constructed and underwent internal and external validation to predict the probability of LNM.

**Results:**

The overall data from the 1517 patients who met the inclusion criteria were analyzed. 105(6.29%) patients had LNM. According the univariate analysis and multivariate logistic regression analysis, LVSI is the most predictive factor for LNM, patients with positive LVSI had 13.156-fold increased risk for LNM (95%CI:6.834–25.324; *P* < 0.001). The nomogram was constructed and incorporated valuable parameters including histological type, histological grade, depth of myometrial invasion, LVSI, cervical involvement, parametrial involvement, and HGB levels from training set. The nomogram was cross-validated internally by the 1000 bootstrap sample and showed good discrimination accuracy. The c-index for internal and external validation of the nomogram are 0.916(95%CI:0.849–0.982) and 0.873(95%CI:0.776–0.970), respectively.

**Conclusions:**

We developed and validated a 7-variable nomogram with a high concordance probability to predict the risk of LNM in patients with EC.

**Supplementary Information:**

The online version contains supplementary material available at 10.1186/s12885-021-08466-4.

## Background

Endometrial cancer (EC) is the most common gynecologic malignancy, especially in developed countries, with the incidence was about 12.9/100,000 [1]. Approximately 70% of EC patients are diagnosed with stage I, and the overall prognosis is favorable, the 5-year survival rate of the patient of stage I was reported ranging from 74 to 91%. However, the metastasis is related to a worse outcome, the 5-year survival rate is 57–66% for stage III, stage IV is only 20–26% [2, 3].

Surgery is the primary intervention for EC which mainly based on total hysterectomy and bilateral salpingo-oophorectomy [4]. Indications for lymphadenectomy remain controversial. Proponents argued that systemic lymph node (LN) resection is necessary for determining the extent of the lesion, accurate staging, directing opportune adjuvant therapy, and it is also a predictive tool for assessing patient prognosis. But this view was challenged by several large-scale clinical randomized controlled trials [5–7], which suggested that patients in the early stage may not get survival benefits from lymphadenectomy. Opponents claimed that with the removal of LN, there is an increased incidence of some complications, such as lymphocyst, lymphoedema, deep vein thrombosis, and intestinal obstruction and so on. Given this, the National Comprehensive Cancer Network (NCCN) guidelines emphasized the importance of assessing risk factors for lymph node metastasis (LNM) preoperatively and intraoperatively, and suggested an individualized and tailored LN dissection way [8].

For the risk assessment of LNM, it is widely accepted that the “Mayo clinic criteria”, low risk refers to endometrioid-type, tumor histology grade I or II, 50% or less myometrial invasion depth, and tumor diameter of 2 cm or less [9]. However, this evaluation criterion was criticized against the accuracy of the frozen section, and it is difficult to achieve uniform quality monitoring.

Nomogram is a graphic calculation tool which has been proposed to visualize and individualize prediction under different situation [10–13]. This study aimed to develop a clinically useful nomogram to predict the LNM in patients with EC by several clinicopathological parameters to help clinicians better screen out high-risk groups and develop appropriate treatment plans.

## Methods

In this retrospective study, a total of 1517 consecutive patients who underwent staging surgery including hysterectomy, pelvic lymphadenectomy (more than 10 LN removed) with or without para-aortic lymphadenectomy for EC were abstracted from January 1st, 2005 to June 31st, 2019 from Qilu Hospital of Shandong University. The current study was approved by the Ethics Committee of Qilu Hospital of Shandong University. All the patients did not receive other treatments such as radiotherapy, chemotherapy or hormones before surgery. Patients with sarcoma, carcinosarcoma, leiomyosarcoma, a double primary tumor, or other metastatic cancer were excluded. Clinicopathological parameters were collected and determined as followed: patient-related characteristics (age at diagnosis, gestation, production, abortion, symptoms before diagnosis including abnormal vaginal fluid and abnormal vaginal bleeding, comorbidities including endocrine and cardiovascular diseases, history of smoking, history of and drinking, menopause), tumor characteristics (histological type, histological grade, FIGO stage, depth of myometrial invasion, lymphovascular invasion (LVSI), cervical involvement, and parametrial involvement) and the results of preoperative hematologic examination (white blood cell (WBC) count, red blood cell (RBC) count, hemoglobin (HGB), blood platelet (PLT), lymphocyte, albumin/globulin ratio(A/G ratio), total cholesterol (TC), and triglyceride). The histological grade and clinical stage were classified according to the 2009 FIGO staging criteria [4].

### Construction and validation of the nomograms

Parameters including patient-related, tumor-related, and preoperative hematologic examination-related were analyzed by univariate analysis. Factors *P* < 0.25 in univariate analysis were included in multivariate logistic analysis. Correlation results were described by the odds ratio (OR) and corresponding 95% confidence interval (CI).

The included population was randomly divided into a training set and a validation set through the software. The training set is used for model construction and internal validation, and the validation set is used for external validation of the model. Based on the multivariate logistic regression analysis results, a nomogram integrating the valuable independent clinicopathological variables was constructed to predict for LNM from the training set. The calibration plot of internal validation was conducted via a bootstrap method with 1000 resamples, by *rms*, a package for R, specifying the parameter “method = “boot“, B =1000”, from the training set (*n* = 1000) [14]. The agreement between the observed outcome and the predicted values was studied using two calibration curves. The receiver operating characteristic (ROC) curve of internal and external validation were plotted, and the area under the curve (AUC) and C-index were calculated to evaluate the accuracy of the prediction. The AUC of internal validation was also calculated via a bootstrap method, by *pROC*, a package for R, specifying the parameter “method = “boot”, B = 1000″, from the training set (*n* = 1000) [14].

### Statistical analysis

All the variables were analyzed by a two-sided statistical test including χ^2^ or Fisher exact test and Student *t* test. Univariate and multivariate logistic regression analyses were performed using SPSS 20. R software package (Version 3.6.2) was used to perform the nomogram and validation of the nomogram. *P* < 0.05 indicates that the difference was statistically significant.

## Results

### Patient and clinical characteristics

The overall data from the 1517 patients who met the inclusion criteria were analyzed. The median age of all patients at the time of surgery was 55 years (range, 21–82 years). 105(6.29%) patients had LNM. Among them, 74(4.87%) patients had pelvic LNM, 5(0.33%) patients had para-aortic LNM, and 26(1.71%) patients had both pelvic and para-aortic LNM. The majority of patients were diagnosed with endometrioid EC (1376/1517,90.70%). We collected some other detailed patient information including symptoms before diagnosis, comorbidities, menstrual history, reproductive history, pathological parameters and several results of preoperative hematologic examinations (Table 1).

**Table 1:** Patient characteristics

	LNM negative	LNM positive	***P value***
	1412	105	
**Age at surgery**			0.002
Mean ± SD	54.42 ± 8.69	57.15 ± 8.05	
Median (range)	55 (21–80)	57 (36–82)	
**Gestation**			0.630
Mean ± SD	2.73 ± 1.46	2.80 ± 1.33	
Median (range)	3 (0–13)	3 (1–6)	
**Production**			0.134
Mean ± SD	1.82 ± 1.13	1.99 ± 0.95	
Median (range)	2 (0–12)	2 (1–5)	
**Abortion**			0.327
Mean ± SD	0.92 ± 1.10	0.81 ± 1.01	
Median (range)	1 (0–7)	1 (1–4)	
**Symptoms**			0.691
Yes	1306	96	
No	106	9	
**Comorbidities**			0.164
Yes	561	49	
No	851	56	
**History of smoking**			0.226
Yes	11	2	
No	1401	103	
**History of drinking**			0.302
Yes	4	1	
No	1408	104	
**Menopause**			0.238
Yes	831	68	
No	581	37	
**FIGO**			
I	1225		
II	113		
III	64	92	
IV	10	13	
**Histologic type**			< 0.001
Endometrioid	1313	63	
Non-endometrioid	99	42	
**Histologic grade**			< 0.001
Well differentiated	651	11	
Moderate/poor differentiated	761	94	
**Depth of myometrial invasion**			< 0.001
<50	1147	41	
≥50	265	64	
**LVSI**			< 0.001
Present	110	37	
Not reported	1302	68	
**Cervical involvement**			< 0.001
Yes	129	31	
No	1283	74	
**Parametrial involvement**			< 0.001
Yes	9	15	
No	1403	90	
**WBC (Mean ± SD,×109/L)**	6.06 ± 1.89	6.74 ± 2.86	0.118
**RBC (Mean ± SD,×109/L)**	4.40 ± 0.49	4.23 ± 0.55	0.001
**PLT (Mean ± SD,×109/L)**	269.29 ± 71.35	283.97 ± 77.62	0.052
**HGB (Mean ± SD, g/l)**	126.29 ± 18.20	119.39 ± 19.96	0.001
**Lymphocyte (Mean ± SD,× 109/L)**	1.94 ± 5.17	1.65 ± 0.59	0.573
**A/G ratio (Mean ± SD)**	1.65 ± 0.28	1.55 ± 0.30	< 0.001
**TC (Mean ± SD, mmol/L)**	5.00 ± 1.00	4.70 ± 0.99	0.004
**Triglyceride (Mean ± SD, mmol/L)**	1.54 ± 1.14	1.29 ± 0.66	0.001

*Abbreviations:LNM* lymph node metastasis, *SD* Standard deviation, *FIGO* International Federation of Gynecology and Obstetrics, *LVSI* lymphovascular invasion, *WBC* white blood cell, *RBC* white blood cell, *PLT* platelet, *HGB* hemoglobin, *A/G ratio* Albumin/globulin ratio, *TC* total cholesterol

### Univariate and multivariate predictors for LNM

According the univariate analysis, age, histological type, histological grade, depth of myometrial invasion, LVSI, cervical involvement, parametrial involvement, HGB, A/G ratio, TC and triglyceride were all significantly associated with LNM, whereas other parameters were not. By multivariate logistic regression analysis, LVSI is the most predictive factor for LNM, patients with positive LVSI had 13.156-fold increased risk for LNM (95%CI:6.834–25.324; *P* < 0.001). In addition, histological type (OR: 3.423; 95%CI: 1.677–6.987; P < 0.001), depth of myometrial invasion (OR: 2.060; 95% CI: 1.073–3.956; *P* = 0.030), cervical involvement (OR: 2.336; 95% CI: 1.117–4.884; *P* = 0.024), parametrial involvement (OR: 7.846; 95% CI: 1.626–37.858; *P* = 0.010) and HGB (OR: 0.983; 95% CI: 0.967–0.999; *P* = 0.039) remained significant predictors of LNM, whereas histological grade was borderline significant (OR: 2.232; 95% CI: 0.915–5.444; *P* = 0.078). Table 2 summarizes the results of the multivariate logistic regression analyses.

**Table 2:** Multivariate logistic regression analysis of the LNM

	OR	95%CI	***P value***
**Histologic type**	3.423	1.677–6.987	< 0.001
**Histologic grade**	2.232	0.915–5.444	0.078
**Depth of myometrial invasion**	2.060	1.073–3.956	0.030
**LVSI**	13.156	6.834–25.324	< 0.001
**Cervical involvement**	2.336	1.117–4.884	0.024
**Parametrial involvement**	7.846	1.626–37.858	0.010
**HGB**	0.983	0.967–0.999	0.039

*Abbreviations*: *LNM* lymph node metastasis, *LVSI* lymphovascular invasion

### Construct a nomogram for the prediction risk of LNM

**Fig. 1:**
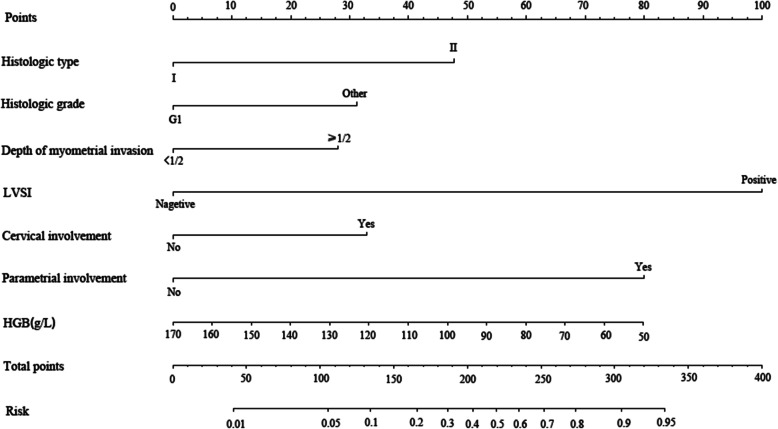
Nomogram predicting the probability of LNM for women with EC

The study group was randomly divided into a training set (1000) and a validation set (517) through the software. The training set is basically consistent with the baseline of the test set (Table 3). As shown in Fig. 1, the nomogram was constructed and incorporated clinical variables from the final multivariate model including histological type, histological grade, depth of myometrial invasion, LVSI, cervical involvement, parametrial involvement, and HGB levels from the training set. For individualized prediction, draw an upward vertical line to the “Points” bar to calculate total points corresponding to the patient’s characteristics. Then, draw a downward vertical line from the “Total Points” line based on the sum to calculate the risk of LNM.

**Table 3:** Patient characteristics between the training set and test set

	training set	test set	***P value***
**N**	1000	517	
**LNM**			0.795
Positive	68	37	
Negative	932	480	
**Age at surgery**			0.450
Mean ± SD	54.73 ± 8.51	54.38 ± 8.99	
Median (range)	55 (27–80)	55 (21–82)	
**Gestation**			0.784
Mean ± SD	2.73 ± 1.47	2.75 ± 1.42	
Median (range)	3 (0–13)	3 (0–8)	
**Production**			0.763
Mean ± SD	1.83 ± 1.16	1.84 ± 1.05	
Median (range)	2 (0–12)	2 (0–7)	
**Abortion**			0.681
Mean ± SD	0.92 ± 1.11	0.89 ± 1.07	
Median (range)	1 (0–7)	1 (0–5)	
**Symptoms**			0.235
Yes	70	45	
No	930	472	
**Comorbidities**			0.816
Yes	400	210	
No	600	307	
**History of smoking**			0.800
Yes	9	4	
No	991	513	
**History of drinking**			0.221
Yes	2	3	
No	998	514	
**Menopause**			0.611
Yes	588	311	
No	412	206	
**FIGO**			0.958
I	811	414	
II	72	41	
III	102	54	
IV	15	8	
**Histologic type**			0.583
Endometrioid	910	466	
Non-endometrioid	90	51	
**Histologic grade**			0.693
Well differentiated	440	222	
Moderate/poor differentiated	560	295	
**Depth of myometrial invasion**			0.987
<50	783	405	
≥50	217	112	
**LVSI**			0.433
Present	122	56	
Not reported	878	461	
**Cervical involvement**			0.934
Yes	105	55	
No	895	462	
**Parametrial involvement**			0.722
Yes	15	9	
No	985	508	
**WBC (Mean ± SD,×109/L)**	6.15 ± 2.04	6.02 ± 1.87	0.225
**RBC (Mean ± SD,×109/L)**	4.39 ± 0.48	4.39 ± 0.52	0.965
**PLT (Mean ± SD,×109/L)**	272.23 ± 71.82	268.09 ± 72.08	0.289
**HGB (Mean ± SD, g/l)**	126.07 ± 18.27	125.31 ± 18.71	0.450
**Lymphocyte (Mean ± SD,× 109/L)**	2.00 ± 6.14	1.75 ± 0.56	0.357
**A/G ratio (Mean ± SD)**	1.63 ± 0.28	1.66 ± 0.28	0.097
**TC (Mean ± SD, mmol/L)**	4.96 ± 0.97	5.00 ± 1.07	0.431
**Triglyceride (Mean ± SD, mmol/L)**	1.54 ± 1.16	1.48 ± 1.03	0.377

*Abbreviations*: *LNM* lymph node metastasis, *SD* Standard deviation, *FIGO* International Federation of Gynecology and Obstetrics, *LVSI* lymphovascular invasion, *WBC* white blood cell, *RBC* white blood cell, *PLT* platelet, *HGB* hemoglobin, *A/G ratio* Albumin/globulin ratio, *TC* total cholesterol

### Accuracy of the nomogram to predict LNM

**Fig. 2:**
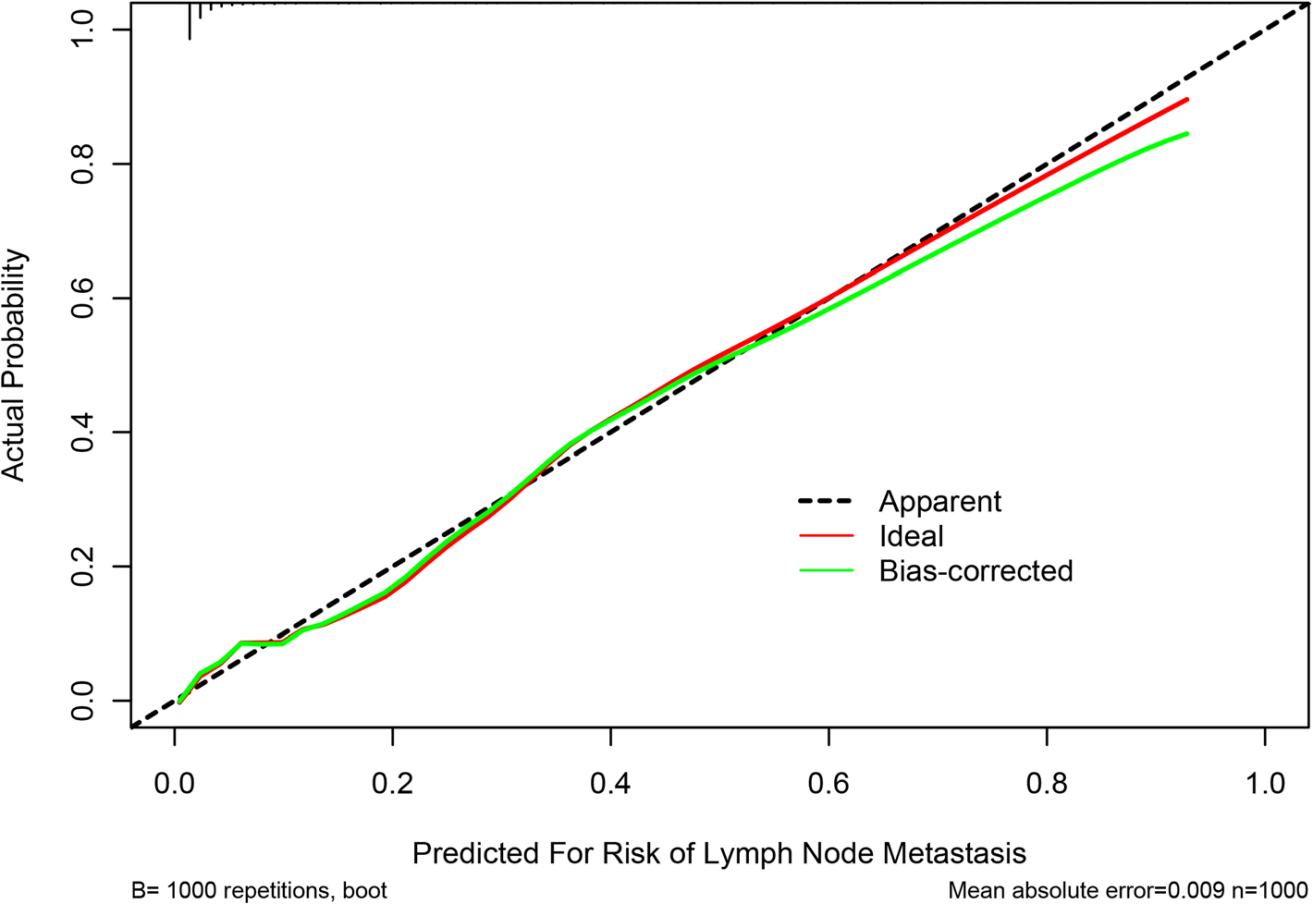
Internal calibration of the nomogram to predict LNM

**Fig. 3:**
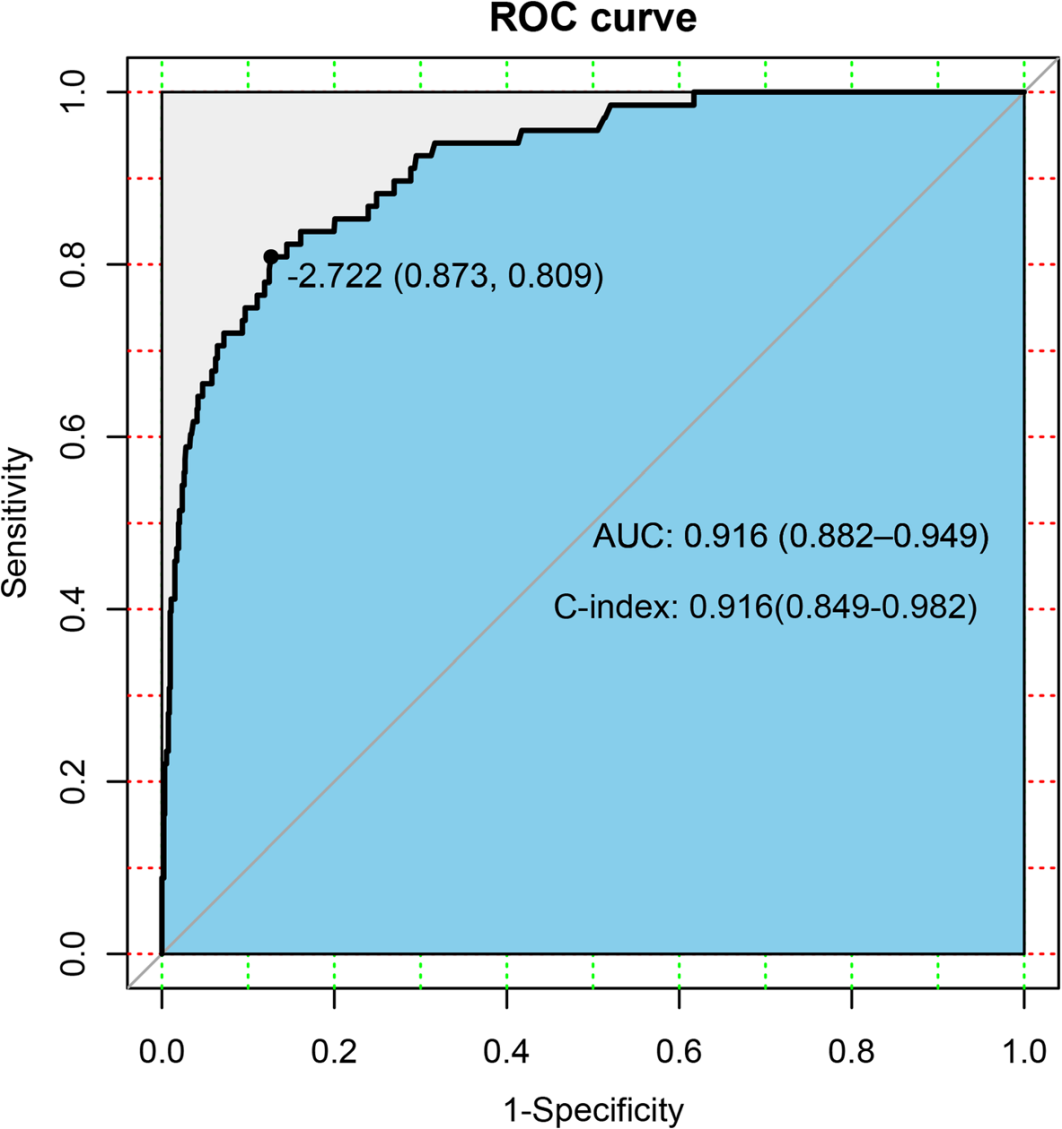
Receiver operating characteristic curves of internal verification corresponding nomogram to predict LNM

**Fig. 4:**
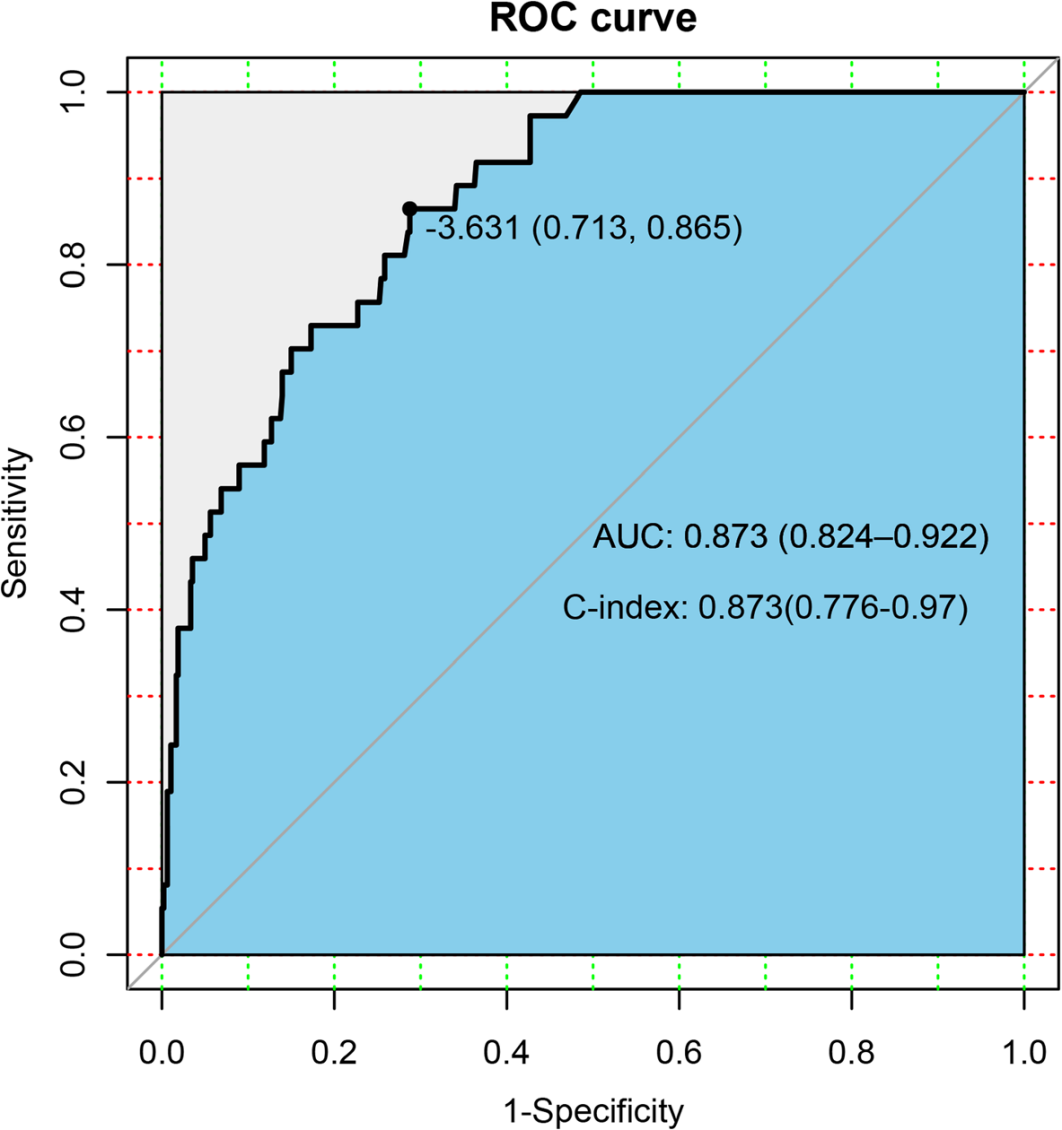
Receiver operating characteristic curves of external verification corresponding nomogram to predict LNM

The nomogram was cross-validated internally by the 1000 repetitions of bootstrap sample corrections. The calibration plots showed in Fig. 2 represents how closely the predictions from the nomogram compared with actual outcomes for the 1000 patients in this study. The value on the X axis represents nomogram prediction and value on the Y axis represents actual probability, while the diagonal dashed line represents the exact match between nomogram prediction and observed probability. For the prediction of LN involvement, the nomogram showed good discrimination accuracy with an AUC of 0.916 (95% CI: 0.882–0.949) and an C-index of 0.916 (95% CI: 0.849–0.982) in internal validation (Fig. 3). In the external validation of the nomogram, the AUC and C-index of the model are respectively 0.873(95% CI:0.824–0.922) and 0.873(95% CI:0.776–0.970) (Fig. 4).

LVSI has a larger proportion in the nomogram that we established, for which the sensitivity analysis was conducted on the prediction model. We used the training set to construct the nomograms without LVSI (Supplementary figure 1), and then carried out internal and external validation (Supplementary figure 2). Subsequently, we found that parametrial involvement had a greater influence in the nomogram without LVSI, and carried out sensitivity analysis in the same way (Supplementary figures 3 and 4). C-indexes of the nomogram without LVSI and the nomogram without LVSI or parametrial involvement were both greater than 0.8. The predictive ability of the nomogram was not greatly affected after sensitivity analysis.

## Discussion

The LN is the most common place for extrauterine metastasis of EC, and the presence of LNM has been demonstrated to be the most important prognostic factor for EC. The risk of LNM account for 3 to 5% in patients with low-grade and superficially invasive EC, while it is approximately 16 to 22% for patients with high-grade disease [9, 15, 16]. The determination of LN status is critical for evaluating prognosis and identifying the necessity of adjuvant therapy. However, the significance of systematic lymphadenectomy remains controversial. There were some large-scale retrospective studies support the therapeutic significance of LN resection, especially for patients with intermediate-high risk factors [17–19]. However, several large-scale clinical randomized controlled trials suggested that patients may not get survival benefits from lymphadenectomy which presumed to be related with increased surgical complications [5, 7]. Thus, we believe that the decision to perform lymphadenectomy should be based on an accurate and individualized risk assessment for LNM.

Multivariate analysis can obtain the coefficient of relevant risk factors, and calculate the specific risk value through the model formula, but it is difficult to integrate the predicted value of these indicators [20, 21]. Recently, research scholars are getting increasingly interested in nomograms [12, 13, 22], which is an intuitive and easily readable graphical chart based on the results by the logistic regression or Cox regression, it could accurately predict the probability of occurrence of an event. For clinical application, the nomogram could incorporate patient individual characteristics and need further validation by cross-validation and bootstrapping methods. In the current study, we constructed a nomogram based on several clinicopathological parameters to predict the risk of LNM. The model may facilitate gynecological oncologists to calculate the incidence of LNM in the individual patient and make a multidisciplinary decision on whether lymphadenectomy is necessary by balancing the risks and benefits. According to the multivariate logistic regression analysis, histological type, histological grade, depth of myometrial invasion, LVSI, cervical involvement, parametrial involvement, and HGB levels are significantly associated with LNM. The brief nomogram was built by the involvement of these seven competing risk models from training set.. Specifically, the nomogram showed good discrimination accuracy with the C-index of 0.916(95%CI:0.849–0.982) of internal validation and an C-index of 0.916(95%CI:0.849–0.982) of internal validation, and a mean error of less than 2% by validation examination in the internal validation, showing excellent predictive performance. What’s more, we carried out sensitivity analysis on the risk prediction model and the predictive ability of the nomogram was fluctuates very little.

LVSI was the most convincing risk predictor for LNM in this study, which is similar to previous studies. Mariani et al. found that adjuvant therapy and lymphadenectomy may be necessary if LVSI was present [23]. Similarly, Pollom et al. proposed an algorithm focusing on pathological and clinical parameters of 296 EC patients, they reported that the positive status of LVSI was significantly associated with LNM [24]. However, Bendifallah et al. developed a nomogram based on the SEER database to evaluate the association of LNM with age, race, histological subtype, histological grade, and depth of myometrium invasion. Nevertheless, the SEER database does not contain information about the patient’s LVSI status, and we presume that the model lacking of LVSI information not comprehensive enough [25].

The determination of LVSI requires evaluation of hematoxylin and eosin (H&E)-stained slides under light microscopy. But it is a challenge for pathologists to determine whether LVSI exists and distinguish it from mimickers such as retraction artifacts. Immunohistochemical staining with CD31, D2–40 and cytokeratin was used to overcome the difficulty of diagnosis [26]. Although it is difficult to determine the presence or absence of LVSI before a hysterectomy, it is still feasible according to the intraoperative frozen section. Previously study showed that there was 92.4% overall agreement between the frozen section and postoperative pathology regarding the presence of LVSI [27]. The limitation of this study is that the LVSI status was evaluated based on the final postoperative pathology. Due to a large number of patients included in the study, we were unable to obtain all the frozen section to determine it intra-operation. But LVSI still has the predictive value especially for incidentally attained patients with EC after hysterectomy.

To be more intuitive and convenient to construct the nomogram, the histological type of EC was classified as endometrioid EC and non-endometrioid EC. And grade differentiation was divided into two categories: well differentiated and moderate/poor differentiated. We found that non-endometrioid EC is a valuable predictor for LNM, which was consistent with previous studies [28]. The special aggressive biological behavior of non-endometrioid EC made it significantly related with worse clinical outcomes. As for tumor grade, it is not considered as a risk factor by the Milwaukee risk stratification model by which lymphadenectomy can be quickly determined through gross examination of tumor diameter and depth of myometrial invasion [29]. However, it was still reported that tumor grade is a significant prognostic factor of EC and an independent predictor for LNM [30]. Our result was consistent with the former study, and we found a positive association between tumor grade and LNM. And we also found that cervical involvement and parametrial involvement were easier to see in patients with LNM, which indicated that the two parameters also have the predictive value for LNM.

The occurrence of malignant tumors is often accompanied by an increased probability of hematological abnormality. It has been demonstrated that systemic immune and inflammation responses play a vital role in the initiation and progression of the malignant tumor [31]. The metabolic diseases such as lipid levels disorders have emerged to be a non-negligible risk factor of EC, and the carcinogenic effect of metabolic abnormality was well established [32, 33]. To further uncover the potential relation between LNM and some hematologic parameters, we collected some detail information including WBC, RBC, HGB, PLT, lymphocyte, A/G ratio, TC and triglyceride. We found that HGB, A/G ratio, TC and triglyceride were all significantly associated with LNM by univariate analysis. However, when combining with other risk factors, A/G ratio, TC and triglyceride were not strong enough to predict LNM. According to the present nomogram, the level of HGB was found to be an independent risk factor in LNM. Our finding was consistent with the former study by Njolstad TS and they found that preoperative anemia was significantly correlated with tumor progression and poor disease-specific survival [34]. The possible explanation may be that the observed anemia caused by vaginal bleeding induced the release of several paracrine signaling factors affecting erythropoiesis, such as the pro-inflammatory cytokines interleukin-1 and tumor necrosis factor-α, which considered to be related with tumor progression and LNM [35].

To the best of our knowledge, this risk prediction model is based on the most comprehensive clinicopathologic parameters and the largest number of included patients in China. Our finding was in line with a dependable nomogram based on some clinical parameters including age, race, tumor grade, histological type, myometrial invasion and cervical stromal invasion, which performed a good discrimination and a reliable calibration to predict LNM [24]. What’s more, the performance of the model fluctuates little and shows good robustness after sensitivity analysis. However, there are still several limitations. First, this is a single-institution study. The application universality and prediction accuracy of the model will be affected by the differences between the tested patients and the model patients. Although bootstrap internal validation was used to mimic new patient cohorts, there is still a need for external validation to ensure the accuracy of the study. Second, most of parameters incorporated in our model can be determined at the frozen section, but the determination of LVSI status can not be judged immediately during surgery. Despite there are defective for predicting intra-operation, it is still helpful for a postoperative decision whether adjuvant therapy or secondary operation was necessary for incidentally attained EC patients. This model also requires a large sample of prospective controlled studies to verify accuracy and utility in the future. It is worth noting that the nomogram model only provides a predictive probability of LNM, the professional interpretation also required according to the individual situation.

## Conclusions

We have developed a 7-variable nomogram with a high concordance probability to predict the risk of LNM in women with EC. The model may facilitate gynecological oncologists to guide clinical individualized treatment plan.

## Supplementary Information

**Additional file 1: Supplementary figure 1.** Nomogram without LVSI predicting the probability of LNM for women with EC.

**Additional file 2: Supplementary figure 2.** Internal verification and external verification of nomogram without LVSI. a. Internal calibration of the nomogram to predict LNM. b. Receiver operating characteristic curves of internal verification. c. Receiver operating characteristic curves of external verification.

**Additional file 3: Supplementary figure 3.** Nomogram without LVSI or parametrial involvement predicting the probability of LNM for women with EC.

**Additional file 4: Supplementary figure 4.** Internal verification and external verification of nomogram without LVSI or parametrial involvement. a. Internal calibration of the nomogram to predict LNM. b. Receiver operating characteristic curves of internal verification. c. Receiver operating characteristic curves of external verification.

## Data Availability

The dataset supporting the conclusions of this article is available in the Qilu Hospital of Shandong University repository, could contact the corresponding author to obtain these data.

## Abbreviations

LN: Lymph Node
EC: Endometrial Cancer
LNM: Lymph Node Metastasis
NCCN: the National Comprehensive Cancer Network
LVSI: Lymphovascular Invasion
WBC: White Blood Cell
RBC: Red Blood Cell
HGB: Hemoglobin
PLT: Blood Platelet
A/G ratio: Albumin/globulin ratio
TC: Total cholesterol
OR: Odds Ratio
CI: Confidence Interval
ROC: Receiver Operating Characteristic
AUC: Area Under the Curve
C-index: Concordance Index
H&E: Hematoxylin and Eosin
