# Supplementary figures and images for: A nomogram prediction model for lymph node metastasis in endometrial cancer patients
Source: BMC Cancer. 2021 Jun 29;21:748. doi: 10.1186/s12885-021-08466-4 (PMC8243766; doi:10.1186/s12885-021-08466-4)

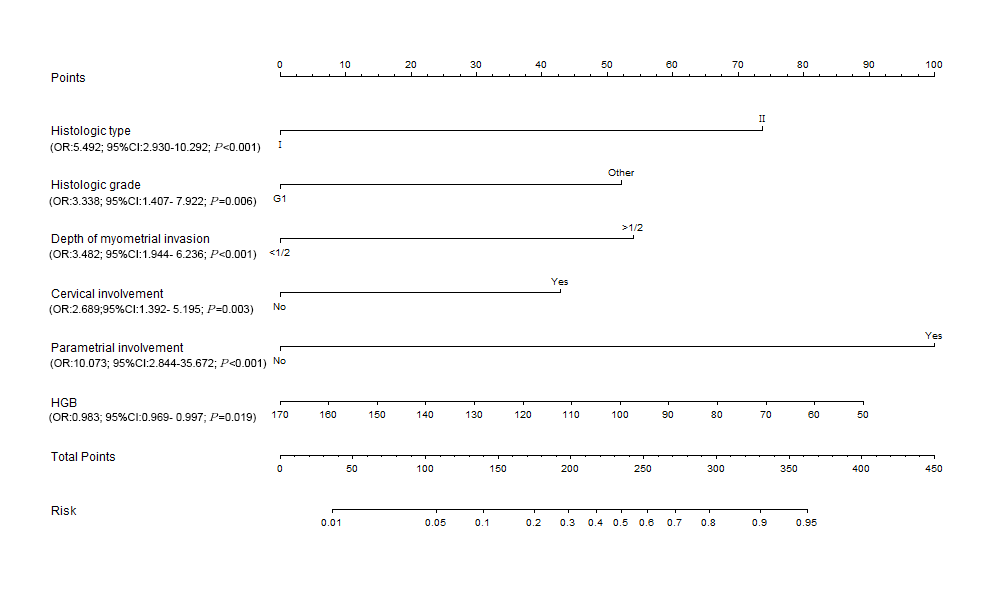

Supplement: Supplementary file 1 — Additional file 1: Supplementary figure 1. Nomogram without LVSI predicting the probability of LNM for women with EC. [file 12885_2021_8466_MOESM1_ESM.tiff]

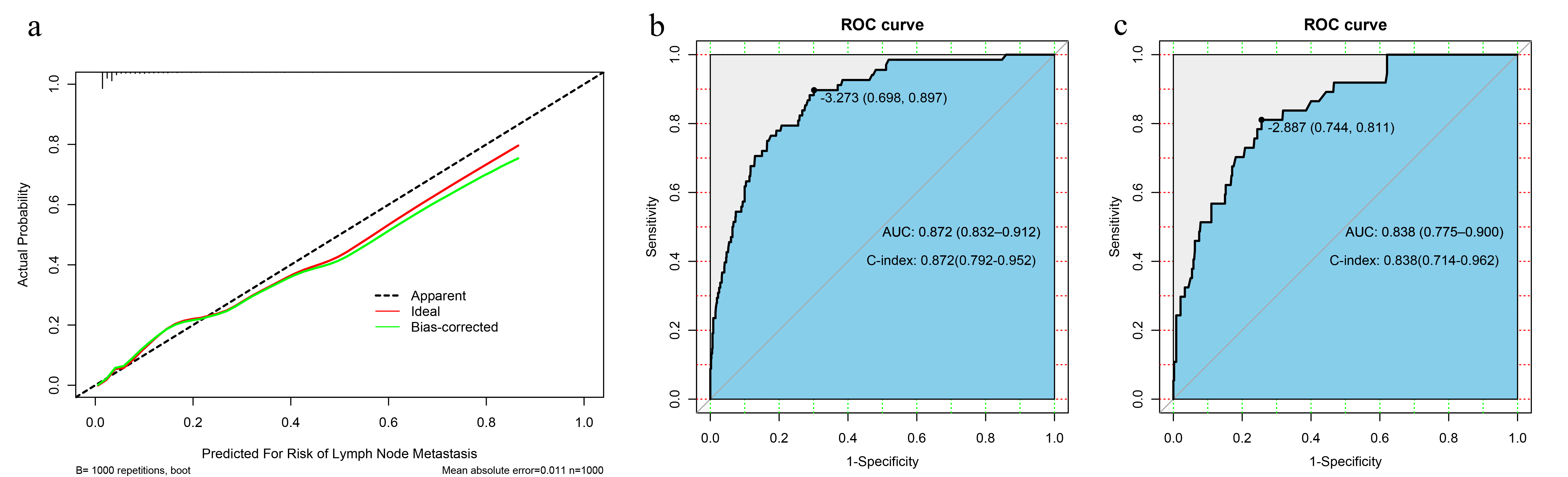

Supplement: Supplementary file 2 — Additional file 2: Supplementary figure 2. Internal verification and external verification of nomogram without LVSI. a. Internal calibration of the nomogram to predict LNM. b. Receiver operating characteristic curves of internal verification. c. Receiver operating characteristic curves of external verification. [file 12885_2021_8466_MOESM2_ESM.tif]

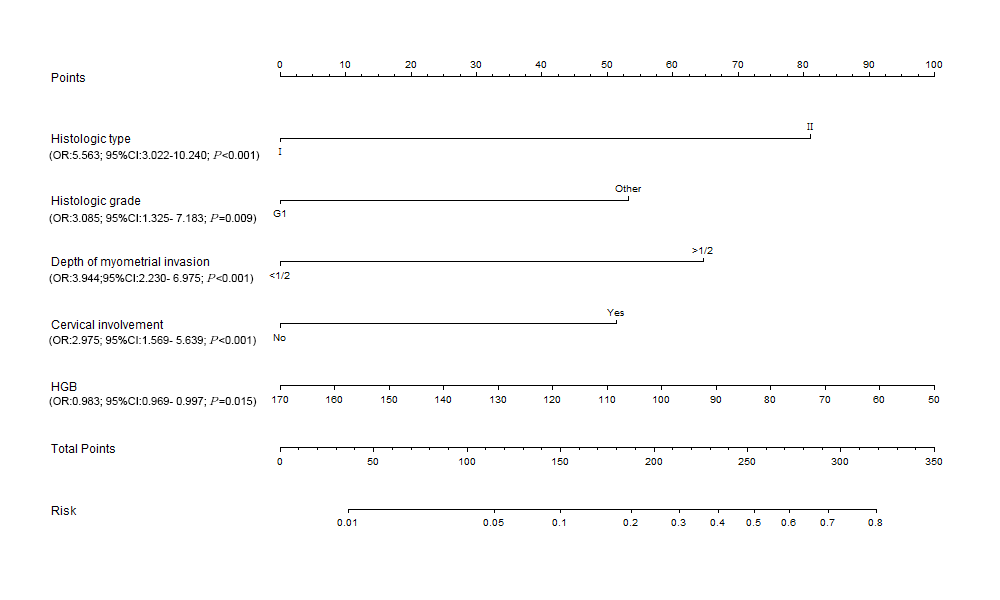

Supplement: Supplementary file 3 — Additional file 3: Supplementary figure 3. Nomogram without LVSI or parametrial involvement predicting the probability of LNM for women with EC. [file 12885_2021_8466_MOESM3_ESM.tiff]

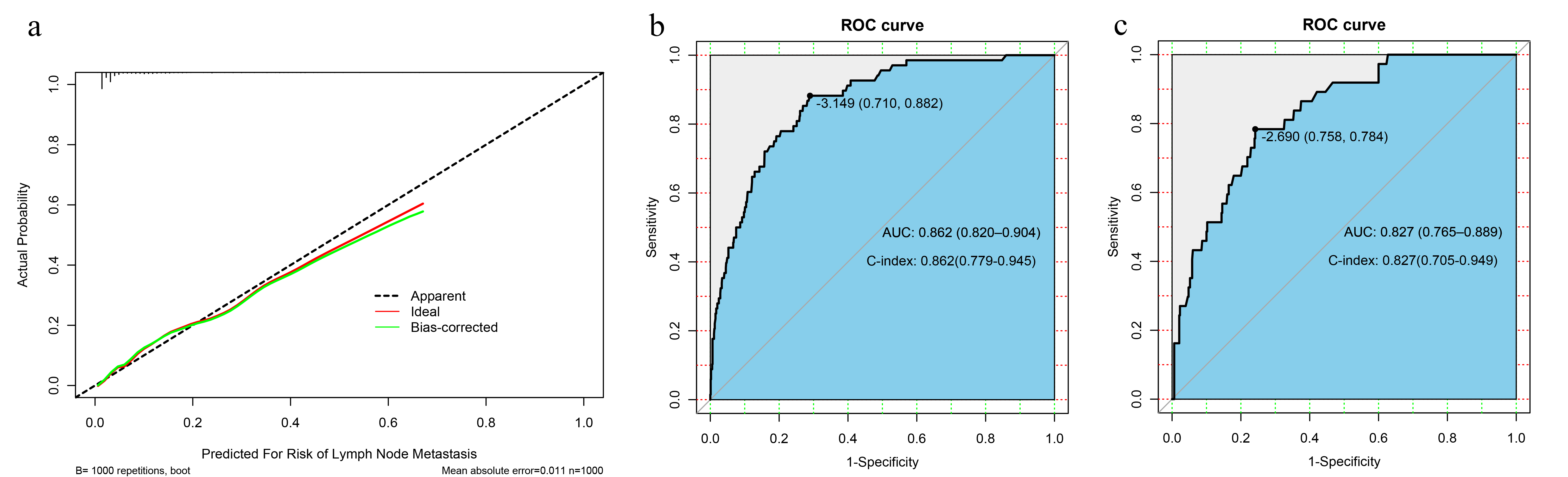

Supplement: Supplementary file 4 — Additional file 4: Supplementary figure 4. Internal verification and external verification of nomogram without LVSI or parametrial involvement. a. Internal calibration of the nomogram to predict LNM. b. Receiver operating characteristic curves of internal verification. c. Receiver operating characteristic curves of external verification. [file 12885_2021_8466_MOESM4_ESM.tif]
